# Morphology and connections of intratrigeminal cells and axons in the macaque monkey

**DOI:** 10.3389/fnana.2013.00011

**Published:** 2013-05-29

**Authors:** Susan Warren, Paul J. May

**Affiliations:** ^1^Department of Neurobiology and Anatomical Sciences, University of Mississippi Medical CenterJackson, MS, USA; ^2^Department of Neurology, University of Mississippi Medical CenterJackson, MS, USA; ^3^Department of Ophthalmology, University of Mississippi Medical CenterJackson, MS, USA

**Keywords:** trigeminal, somatosensory, face, oro-facial reflexes, blink

## Abstract

Trigeminal primary afferent fibers have small receptive fields and discrete submodalities, but second order trigeminal neurons often display larger receptive fields with complex, multimodal responses. Moreover, while most large caliber afferents terminate exclusively in the principal trigeminal nucleus, and pars caudalis (sVc) of the spinal trigeminal nucleus receives almost exclusively small caliber afferents, the characteristics of second order neurons do not always reflect this dichotomy. These surprising characteristics may be due to a network of intratrigeminal connections modifying primary afferent contributions. This study characterizes the distribution and morphology of intratrigeminal cells and axons in a macaque monkeys. Tracer injections centered in the principal nucleus (pV) and adjacent pars oralis retrogradely labeled neurons bilaterally in pars interpolaris (sVi), but only ipsilaterally, in sVc. Labeled axons terminated contralaterally within sVi and caudalis. Features of the intratrigeminal cells in ipsilateral sVc suggest that both nociceptive and non-nociceptive neurons project to principalis. A commissural projection to contralateral principalis was also revealed. Injections into sVc labeled cells and terminals in pV and pars oralis on both sides, indicating the presence of bilateral reciprocal connections. Labeled terminals and cells were also present bilaterally in sVi and in contralateral sVc. Interpolaris injections produced labeling patterns similar to those of sVc. Thus, the rostral and caudal poles of the macaque trigeminal complex are richly interconnected by ipsilateral ascending and descending connections providing an anatomical substrate for complex analysis of oro-facial stimuli. Sparser reciprocal crossed intratrigeminal connections may be important for conjugate reflex movements, such as the corneal blink reflex.

## Introduction

Studies in a variety of mammalian species have identified the locations of the trigeminal neurons contributing to a network of intratrigeminal connections between the subnuclei of the trigeminal complex: the principal nucleus (pV), the spinal trigeminal nucleus pars oralis (sVo), pars interpolaris (sVi), and pars caudalis (sVc) in several non-primate species (rat: Falls, 1984a,b; Jacquin et al., 1990b; Voisin et al., 2002; Timofeeva et al., 2004; cat: Carpenter and Hanna, 1961; Stewart and King, 1963; Hockfield and Gobel, 1978, 1982; Panneton and Burton, 1982; Ikeda et al., 1984; Nasution and Shigenaga, 1987; sheep: Roberts and Matzke, 1971). Even the mesencephalic trigeminal neurons send axon collaterals that terminate within the principal and spinal trigeminal nuclei (rat: Luo et al., 1995; cat: Shigenaga et al., 1990), and this nucleus may receive ascending input from the spinal trigeminal nucleus (Buisseret-Delmas et al., 1997). These studies have tended to focus on the pattern of ascending connections originating in sVc, and have suggested that the ascending pathway could provide a circuit for modifying the activity of pV and/or sVo neurons. The physiology and functions of the trigemino-trigeminal connections have not been extensively investigated. In cat intracellular studies, intratrigeminal neurons in sVc were shown to fall into both nociceptive and non-nociceptive categories (Hu et al., 1981). Similarly, cells with intratrigeminal axons in the rat's sVo and sVi include cells with a wide variety of physiological and anatomical types (Jacquin et al., 1989; Jacquin and Rhoades, 1990).

In humans, trigeminal sensation is critical to proper speech formation, and higher primates lack the distinctive specializations of animals who possess vibrissae. However, only a few early studies have investigated the intratrigeminal pathways in primates (marmoset: Dunn and Matzke, 1968; squirrel monkey and baboon: Tiwari and King, 1974; squirrel monkey: Ganchrow, 1978). In view of the morphological and physiological data obtained in non-primates, a systematic examination of the interconnections between the subnuclei of the macaque trigeminal complex with contemporary tracer techniques seemed warranted. In order to characterize the pattern of intratrigeminal axonal projections, as well as identify the neurons that give rise to these connections, pressure injections of wheat germ agglutinin conjugated horseradish peroxidase (WGA-HRP) or biotinylated dextran amine (BDA) were placed into either sVc, sVi, or pV of macaque monkeys. Parts of this work have been presented in abstract form (Warren et al., 1997).

## Materials and methods

The experiments described in this report were conducted in accordance with PHS Policy on *Humane Care and Use of Laboratory Animals* and the NIH *Guide for the Care and Use of Laboratory Animals*. All the procedures were approved by the Institutional Animal Care and Use Committee at the University of Mississippi Medical Center. Material from 19 adult macaque monkeys (*Macaca fascicularis* and *Macaca mulatta*) of both sexes, ranging from 3.0 to 5.8 kg in weight, was used in this study. Most of these animals were also utilized in other non-conflicting experiments. In order to anterogradely label the intratrigeminal fibers, and to retrogradely label the cells of origin of this pathway, pressure injections of BDA, 10,000 mW (Molecular Probes, Inc., Eugene, OR, USA) were placed in either pV (*N* = 3), sVc (*N* = 3), or sVi (*N* = 4). WGA-HRP (Sigma-Aldrich, St. Louis, MO, USA) injections were placed into pV (*N* = 2) or sVc (*N* = 2). We analyzed five additional control cases. In these animals, either injections of BDA were made into: the middle cerebellar peduncle, pontine reticular formation or medulla medial to sVc, or injections of WGA-HRP were made into the spinal cord medial to sVc.

## General surgical procedures

For all surgical procedures, animals were sedated with ketamine HCl (10 mg/kg, IM) (Ben Venue Laboratorie, Inc., Bedford, OH, USA). They were then anesthetized with Isoflurane (1-3%) (Baxter Health Corp., Deerfield, IL, USA) administered via a tracheal tube before placement in a stereotaxic apparatus. Core body temperature and vital signs were monitored and maintained within normal ranges. Hydration was maintained with an intravenous drip. Dexamethasone (1.0 mg/kg, IV) (Bimeda-MTC Health Corp., Cambridge, ON, Canada) was used to control CNS edema and atropine sulfate (0.05 mg/kg, IM) reduced tracheal secretions.

For injections directed at pV, animals received a unilateral craniotomy. Following the craniotomy, the dura mater was incised and reflected, and a portion of the medial parietal cortex between the central sulcus and the intraparietal sulcus was aspirated to reveal the tectum and anterior edge of the tentorium cerebelli. The latter was incised to reveal the dorsolateral surface of the cerebellum, and the caudolateral surface of the midbrain. The cerebellum was gently retracted to reveal the exiting trochlear nerve, which marked the point along the junction of the middle cerebellar peduncle and pontine tegmentum that overlies pV. The needle of a 5 μl Hamilton microsyringe, angled 2° tip medial in the coronal plane, was positioned at the peduncle-tegmentum junction, and lowered 4.0–4.5 mm to reach the pV. In some cases (*N* = 2), the face was stimulated with a swab to locate a field potential. Several small injections were made along the trajectory of a single penetration, in an attempt to fill the pV. The injection consisted of either a 10% solution of BDA, with a total of 0.1–0.5 μl injected, or a 2.0% solution of WGA-HRP with 0.02–0.04 μl injected. The aspiration defect was filled with hydrated gelfoam. The incision was closed in layers. Animals received buprenorphine (0.01 mg/kg/12 h, IM) (Reckitt Benckiser Pharmaceuticals Inc., Richmond, VA, USA) in the initial 24 h postoperative period.

To approach the caudal end of the spinal trigeminal nucleus, the animal was positioned in the stereotaxic apparatus with its head inclined, nose-down, 25–35°, and its body raised on a platform to limit traction on the spinal cord. A midline scalp incision extended from the external occipital protuberance to the C_3_ vertebra, and the underlying neck muscles were incised along the ligamentum nuchae and reflected laterally. The head was further inclined to a final position of 40 to 45° nose-down, to moderately flex the cranio-cervical junction. A portion of the C_1_ vertebral arch was removed and the atlanto-occipital membrane was reflected to visualize the dorsal medulla to the level of the obex. Using surface landmarks, the needle of a 1 μl Hamilton microsyringe was positioned over the tuberculum cinerium. The needle was subsequently lowered into the sVc. Approximately 0.2 μl of 10% BDA or 0.05 μl of 2.0% WGA-HRP solution was injected along each track. Several tracks, varying only in their rostral-caudal position were made, in an attempt to better fill this long nucleus. When sVi was the target, the syringe tip was angled rostrally, and the entrance point moved closer to the level of the obex. Following the injections, the defect in the atlanto-occipital membrane was closed with Gelfilm, and the incision was closed in layers. The postoperative treatment was the same as for the pV cases.

After allowing a transport time of 14–21 days for BDA and 1–2 days for WGA-HRP, the animals were euthanized by an overdose of sodium pentobarbital (50 mg/kg, IP) (Ludbeck Inc., Deerfield, IL, USA). They were perfused transcardially with a 0.9% saline prewash followed by a double aldehyde fixative (1% paraformaldehyde and 1.25–1.5% glutaraldehyde) in a 0.1 M sodium phosphate buffer (PB) (pH 7.2). The brain was blocked in the frontal plane *in situ*, and tissue blocks, including the brainstem, were harvested.

## Histochemistry and analysis

Brainstem blocks were sectioned at 100 μm in the frontal plane using a Vibratome, and retained in serial order. To reveal the BDA reaction product, the tissue sections were processed using an Avidin-HRP protocol (Wang et al., 2010). Briefly, the tissue was rinsed in 0.1 M PB (pH 7.2) containing 0.05–0.1% TritonX-100. Sections were then incubated overnight at 4°C with gentle agitation in a 10% Avidin-HRP (Vector 1:5000) solution in 0.1 M PB (pH 7.2) containing 0.05–0.1% TritonX-100. After rinsing with 0.1 M PB (pH 7.2), the tissue was reacted in diaminobenzidine (DAB) (1.0%) containing 0.001% hydrogen peroxide with 0.001% nickel ammonium sulfate and 0.001% cobalt chloride in 0.1 M PB (pH 7.2) for 10–30 min. Sections were rinsed with 0.1 M PB (pH 7.2), and were then mounted on gelatin coated slides, counterstained with cresyl violet, cleared, and coverslipped.

To reveal the WGA-HRP, sections were reacted using a tetramethylbezidine (TMB) protocol (Olucha et al., 1985). Briefly, the tissue was rinsed with 0.1 M PB, (pH 6.0). The sections were incubated with 0.005% TMB (tetramethylbenzidine HCl), 0.25% ethanol, and 0.245% ammonium molybdate in 0.1 M PB (pH 6.0). The reaction was initiated by the addition of hydrogen peroxide solution (0.011%), and the sections incubated overnight at 4°C with gentle agitation. Sections were transferred to a stabilizer solution containing 5.0% ammonium molybdate in 0.1 M PB (pH 6.0). They were then rinsed with 0.1 M PB (pH 6.0), and prepared for microscopy as described above.

The distributions of both anterogradely labeled axons and retrogradely labeled neurons within the brainstem trigeminal complex were charted using an Olympus BH2 microscope equipped with a drawing tube. The morphology of selected neurons and axonal arbors was drawn at 320× by use of a 100× oil objective. Measurements of the long axis dimension were made on these drawings for 25 cells for each subnucleus of the trigeminal sensory complex (excluding contralateral pV following sVc injections, where the retrograde labeling was sparse) from the case for each injection type with the best cell labeling. A Nikon Eclipse E600 photomicroscope equipped with a Nikon digital DS-Ri1 camera was used to photograph the material. These images were acquired with the Elements (Nikon) image analysis software. Digitized information from up to 10 Z-axis focal planes was combined into a single plane. The brightness, contrast, and color of the digitized images were adjusted in Adobe Photoshop to appear as close as possible to the visualized image.

## Results

### Intratrigeminal labeling following principal nucleus injections

Following injections of WGA-HRP or BDA into the principal trigeminal sensory nucleus (pV), the distribution and morphology of labeled neurons and axons was characterized. We always observed excellent anterograde filling of axons with BDA, but robust retrograde filling of the neurons at greater distances from the injection site was only found with larger injections of BDA. Consequently, we have illustrated examples of these larger injections. Efforts were made to confine injections to pV, however, tracer often spread to involve the rostral sVo. Furthermore, specifying the precise location of the border that separates pV from sVo proved difficult. Therefore, we have grouped these nuclei together, and referred to the injected region as pV/sVo.

**Figure 1:**
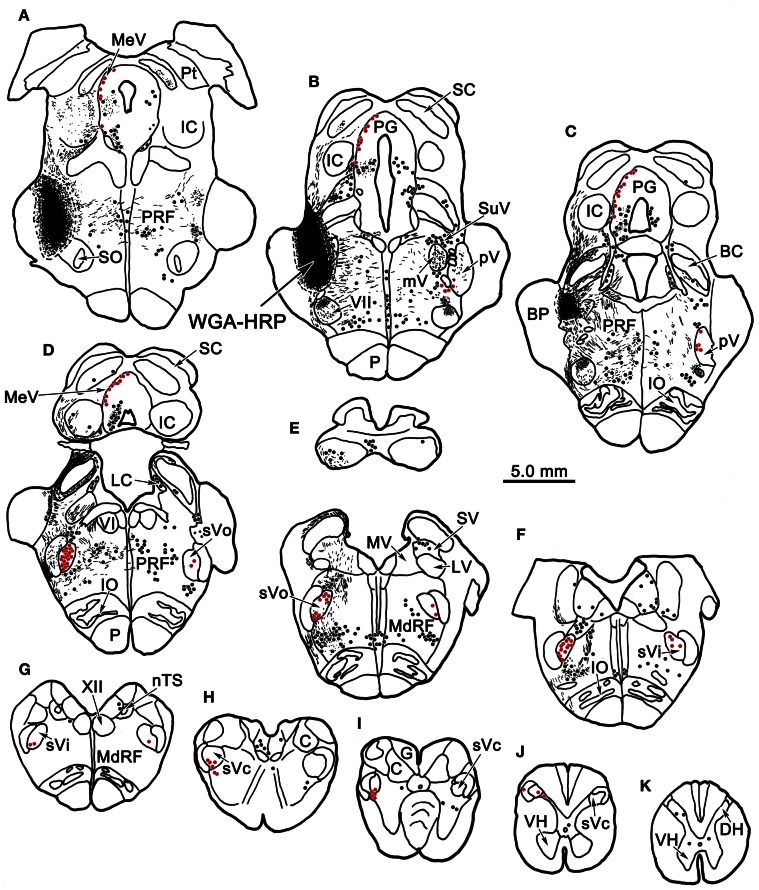
**Charting of the distribution of anterogradely labeled axons (lines) and terminals (stipple), and retrogradely labeled neurons (dots) following a wheat germ agglutinin conjugated horseradish peroxidase (WGA-HRP) injection centered in the principal trigeminal nucleus.** Labeled intratrigeminal neurons (red dots) and terminals are distributed throughout the spinal trigeminal complex. Other retrogradely labeled neurons (black dots) were concentrated in the reticular formation and periaqueductal gray, and were also present in vestibular nuclei and spinal cord. Abbreviations for figures: BC, brachium conjunctivum; BP, brachium pontis; C, cuneate nucleus; Cf, cuneate fasciculus; DH, dorsal horn; DV, descending vestibular nucleus; eC, external cuneate nucleus; G, gracile nucleus; Gf, gracile fasciculus; IC, inferior colliculus; IO, inferior olive; LC, locus coeruleus; LV, lateral vestibular nucleus; MdRF, medullary reticular formation; MeV, mesencephalic trigeminal nucleus; mV, motor trigeminal nucleus; MV, medial vestibular nucleus; nTS, nucleus tractus solitarius; P, pyramid; PG, periaqueductal gray; PRF, pontine reticular formation; Pt, pretectum; pV, principal trigeminal nucleus; SC, superior colliculus; SO, superior olive; SpTT, spinal trigeminal tract; SuV, supratrigeminal area; SV, superior vestibular nucleus; sVc, pars caudalis of the spinal trigeminal nucleus; sVi, pars interpolaris of the spinal trigeminal nucleus; sVo, pars oralis of the spinal trigeminal nucleus; VH, ventral horn; VI, abducens nucleus; VII, facial nucleus; XII, hypoglossal nucleus; 5 nm, motor division of the trigeminal nerve; 5 ns, sensory division of trigeminal nerve; 7n, facial nerve.

Figure 1 plots the distribution of anterogradely labeled axon terminals (stipple) and retrogradely labeled neurons (red dots) in the trigeminal nuclei following an injection of WGA-HRP into the left pV/sVo (Figures 1A–C). WGA-HRP was also present along the needle track as it pierced the lateral portion of the parabrachial nuclei (Figures 1B–D). Anterogradely labeled axon terminals were sparse and restricted to the dorsomedial region of sVo, ipsilaterally (Figures 1D,E). Labeled axon terminals were also seen within the trigeminal motor nucleus contralaterally (Figure 1B) and in the facial motor nucleus bilaterally (Figures 1B,C). At the level of the injection, retrogradely labeled cells were located within the contralateral pV (Figures 1B,C). Retrogradely labeled neurons were present bilaterally in sVo (Figures 1D,E), with an ipsilateral predominance. Within the ipsilateral sVi (Figures 1F,G), retrogradely labeled neurons were located mainly within more rostral regions of the nucleus. Retrogradely labeled neurons were most common in the superficial laminae (I–III) of the ventrolateral region of sVc, ipsilaterally (Figures 1H–J). There was a near absence of labeled neurons in the contralateral sVc (Figures 1H–J). Retrogradely labeled cells were found outside the trigeminal nucleus (black dots) in the periaqueductal gray, reticular formation, vestibular nuclei, and spinal cord.

**Figure 2:**
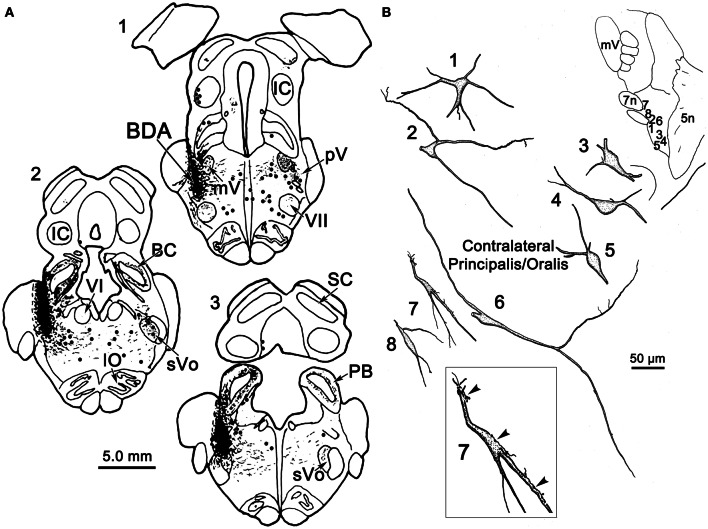
**(A)** Shows a BDA injection into the left pV and sVo, with retrogradely labeled cells (dots), axons (lines), and terminals (stipple) indicated. **(B)** The morphology of retrogradely labeled cells and axons found in the contralateral pV/sVo following the injection. Their location is shown in the insert (upper right). The box shows a higher magnification view of Cell 7, revealing close associations (arrowheads) between labeled axonal boutons and this cell.

**Figure 3:**
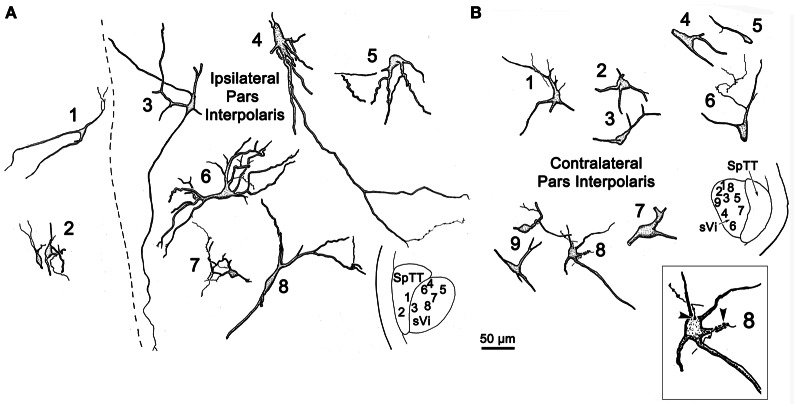
**Retrogradely labeled neurons and anterogradely labeled axons within sVi on the ipsilateral (A) and contralateral (B) side following the injection illustrated in Figure 2A.** The location of the illustrated neurons is shown in the low magnification inserts. Close associations (arrowheads) between a labeled cell (Cell 8) and axonal boutons on the contralateral side are shown in the box.

**Figure 4:**
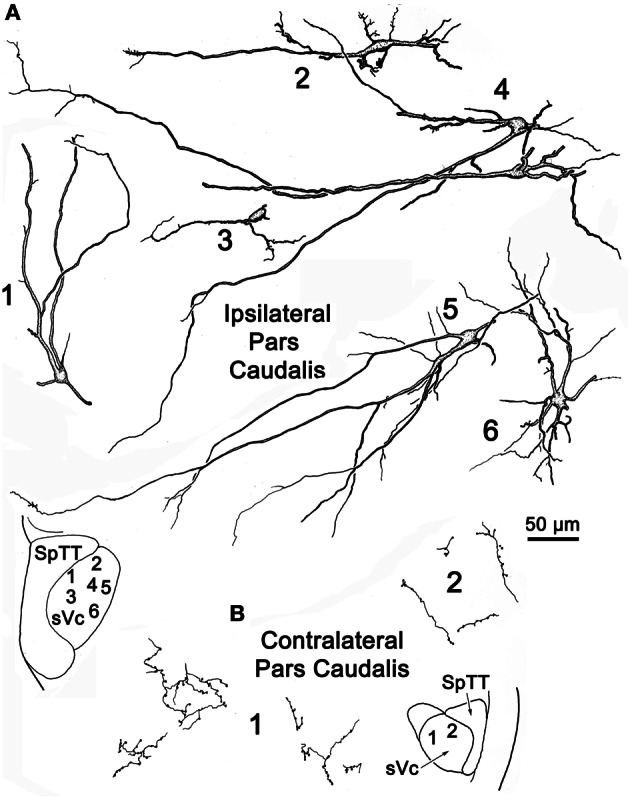
**Retrogradely labeled neurons and anterogradely labeled axons within the sVc on the ipsilateral (A) and contralateral (B) side following the injection illustrated in Figure 2A.** Labeled intratrigeminal neurons were only seen ipsilaterally **(A)**, not contralaterally **(B)**. The location of the illustrated neurons is shown in the low magnification inserts.

Figure 2A illustrates a BDA injection centered in the left pV with limited spread into the rostral portion of sVo (Figure 2A_1–3_). BDA was also present along the needle track as it pierced the lateral portion of the parabrachial nuclei. Like the WGA-HRP injection, this injection produced labeled neurons within the sVi and sVc, ipsilaterally, as well as pV, sVo, and sVi, contralaterally. Anterogradely labeled axon terminals were noted in sVi and sVc ipsilaterally, and in pV, sVi, and sVc, contralaterally. The morphology of individual labeled neurons and labeled axon terminals produced by this injection are illustrated in Figures 2B, 3 and 4. However, we did not analyze ipsilateral anterograde labeling because it likely included labeled primary afferents.

Examples of the morphology of labeled neurons within the contralateral pV/sVo are displayed in Figure 2B. The labeled neurons were most common in the ventral and medial portion of pV, adjacent to the exiting facial nerve fibers. Although found throughout the contralateral pV, labeled axon terminals were densest in the portion of pV just lateral to the exiting fibers of the trigeminal and facial motor nuclei. Both labeled neurons and terminals were observed to cross into the rostral portion of sVo. The dendritic branches of these cells were not completely filled, but appeared to branch very little. The cell somata were mainly medium sized (long axes = 24–35 μm). Many of the somata were fusiform in shape (Cells 6, 7, and 8), but others were multipolar (Cells 1, 2, and 3). Cell 7 displays a labeled axon in close association with its dendrite (insert box). The proximity of the retrogradely labeled neuron and the boutons of this anterogradely labeled axon suggest commissural neurons in pV may be reciprocally connected.

Figure 3 illustrates the morphology of labeled neurons and axon terminals found in sVi following a pV injection (Figure 2A). BDA labeled neurons were distributed throughout the ipsilateral sVi and displayed extensive labeling of their dendrites. The labeled cells within ipsilateral sVi (Figure 3A) had fusiform, and more commonly, multipolar somata. Scattered smaller neurons (long axes around 20 μm), with relatively simple dendritic fields, were found within the spinal trigeminal tract (SpTT) itself (Cells 1 and 2). These may lie within the paratrigeminal nuclei (Saxon and Hopkins, 1998). Within the nucleus proper, the somata size varied considerably (long axes = 13–46 μm). Some cells had relatively simple dendritic fields, produced by sparsely branching dendrites (Cells 3 and 8), while the dendrites of other cells displayed more complex branching (Cells 4 and 6). There was no obvious pattern of overall dendritic orientation. However, long dendrites were often observed running parallel to SpTT (Cell 3).

Compared to the labeled intratrigeminal neurons within ipsilateral sVi (Figure 3A), the dendritic branches of contralateral sVi neurons (Figure 3B) were less completely filled, but still distinctly multipolar. Their soma size was similar to those in the ipsilateral sVi, ranging from small (long axes = 13–20 μm) (Cells 3, 5, and 9) to medium (long axes = 21–33 μm) (Cells 1, 2, 4, 6, 7, and 8) in size. Most were distributed within the central core of sVi. Several labeled sVi neurons (Cells 1, 6, and 8) displayed close associations (arrowheads) with anterogradely labeled axons, as best demonstrated in the insert for Cell 8. The labeled axons in sVi had few branches and so primarily displayed *en passant* swellings.

Figure 4 illustrates labeled neurons and axonal arbors distributed within ipsilateral and contralateral sVc (Figures 4A,B, respectively) following a pV injection (Figure 2A). The somata of these cells varied considerably in size (long axes = 14–29 μm). Intratrigeminal neurons were particularly common in lamina IV of ipsilateral sVc (Cells 2, 4, and 5). These displayed morphologies reminiscent of cortical pyramidal cells. Their branched dendrites extended into lamina I. Other labeled cells, like Cell 1, were located adjacent to the SpTT. Their sparsely branched dendrites were oriented within the lamina. Similarly, Cell 6, located close to the adjacent reticular formation, displayed more highly branched dendrites that mainly ran parallel to the nuclear border. Small neurons like Cell 3 were less common. These smaller cells had circumscribed, simple dendritic fields. The contralateral sVc lacked retrogradely labeled neurons. As shown in Figure 4B, this subnucleus contained only fine, anterogradely labeled axon terminals with *en passant* and terminal puncta similar to those observed in contralateral sVi.

**Figure 5:**
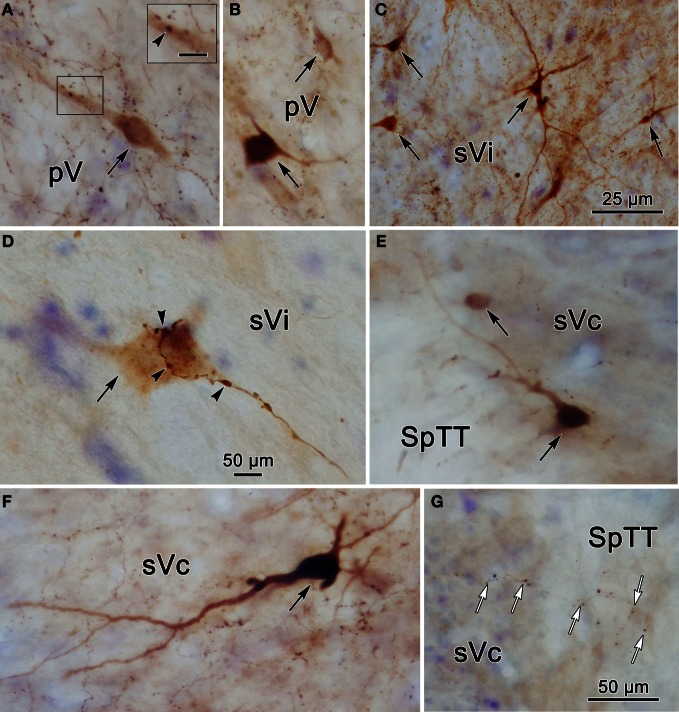
**Photomicrographs of labeled neurons (black arrows) and axons within the trigeminal complex following the injection illustrated in Figure 2A.** Panels **(C,E,F)** are from the ipsilateral side and panels **(A,B,D,G)** are from the contralateral side. Panels **(C)** and **(D)** are low and high magnification photomicrographs, respectively, of cells in sVi. Close associations (arrowheads) between labeled boutons and a labeled cell are shown in **(D)**. The box in **(A)** shows a higher magnification view where close associations (arrowheads) are present. Panels **(E–G)** are from sVc. White arrows in **(G)** indicate labeled axonal boutons. Scale in the boxes is 50 μm. Z axis planes used to produce plate: **(A)** = 1, **(B)** = 2, **(C)** = 1, **(D)** = 1, **(E)** = 4, **(F)** = 3, **(G)** = 4. Scale in **(G)** for **(B–D)** and **(F)**.

To provide further information on the morphology of the labeled cells and fibers photomicrographs of labeled elements found in the trigeminal nuclei following a unilateral injection into pV (Figure 2A) are presented in Figure 5. Labeled neurons within contralateral pV have medium sized somata with fusiform or multipolar morphologies (Figures 5A,B). Evidence of a close association (arrowhead) between a commissural axon terminal and a labeled neuron is shown in the insert (Figure 5A). Figure 5C demonstrates the variety of labeled neurons within ipsilateral sVi. Similar, but far fewer labeled cells were present in the contralateral sVi (Figure 5D), and close associations (arrowheads) between labeled neurons and axon terminals were present. The labeled neurons in ipsilateral sVc were dominated by cells in lamina IV, which had a pyramidal appearance (Figure 5F) and dendrites that extended to lamina I. Cells in lamina I with dendrites extending parallel to the lamina were also present (Figure 5E). Figure 5G reveals the appearance of fine, labeled axon terminal arbors (white arrows) within the contralateral sVc that displayed small boutons.

### Intratrigeminal labeling following spinal trigeminal nucleus injections

**Figure 6:**
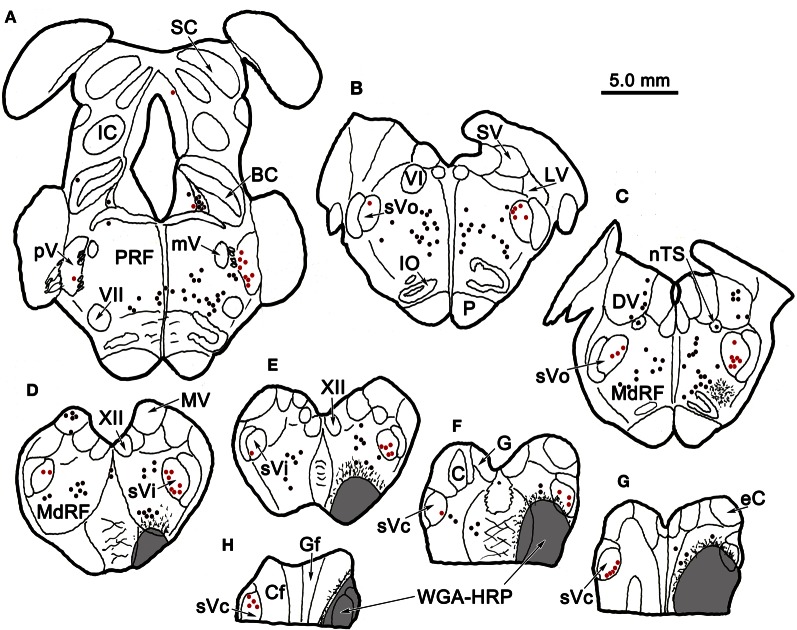
**Distribution of retrogradely labeled neurons in the trigeminal nuclear complex (red dots) and the brainstem (black dots) following an injection of WGA-HRP into sVc (F–H) and the adjacent reticular formation (E–H).** Note the contralateral label in sVc **(F–H)** and the bilateral label in sVi, sVo, and in pV, as well as the non-trigeminal label in the reticular formation and vestibular nuclei.

Figure 6 charts the distribution of retrogradely labeled cells following an injection of WGA-HRP into sVc (Figures 6F–H) at the spinomedullary junction. The injection also included the SpTT and the medullary reticular formation immediately ventral, lateral, and rostral to sVc. The SpTT displayed few labeled axons, in agreement with the limited fiber of passage uptake of WGA-HRP. Retrogradely labeled neurons were observed bilaterally within the trigeminal sensory complex (red dots). They were present contralaterally within sVc (Figures 6F–H) and bilaterally within sVi (Figures 6D,E), but most of these cells were ipsilateral. They were also present bilaterally in sVo (Figures 6B,C) and in pV (Figure 6A), although very few cells were seen in contralateral pV. Retrogradely labeled cells were also present outside the trigeminal nuclei (black dots), particularly in the pontine and medullary reticular formation, and the vestibular nuclei. These were most likely due to the spread of the tracer into the reticular formation adjacent to sVc. We examined two additional cases with WGA-HRP injections to confirm this case (not illustrated). In one case, the injection site spread from C_1−3_ and included sVc, plus the adjacent dorsal quadrant of the spinal cord. In the second case, the dorsal quadrant was injected, but the tracer did not include sVc. The pattern of retrograde label in the trigeminal complex described above was only seen when the tracer included sVc.

**Figure 7:**
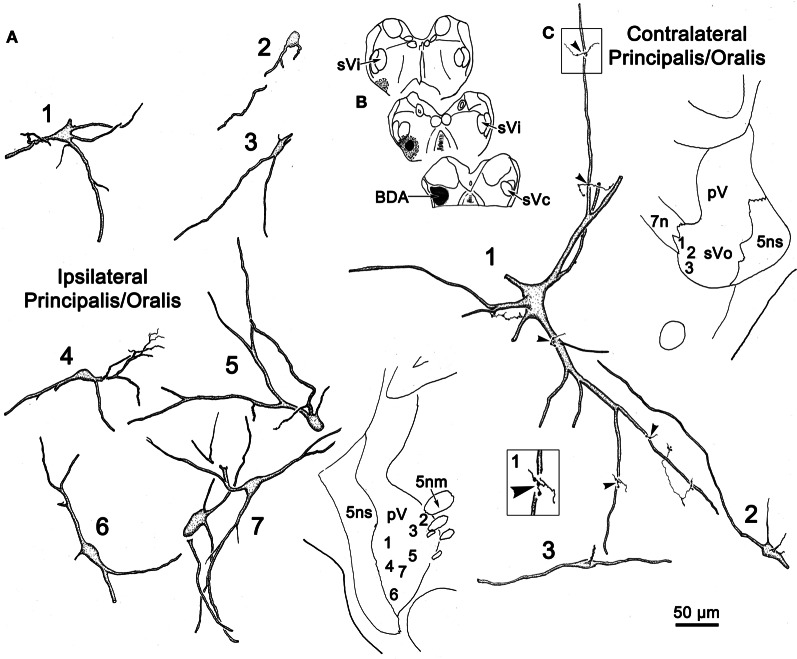
**A unilateral injection into sVc is shown in (B). (A,C)** illustrate the morphology of the cells labeled following this injection, which were seen in the border region between pV and sVo on the ipsilateral **(A)** and contralateral **(C)** side. Labeled axon terminals were seen in close association (arrowheads) with labeled cells, as shown in the higher magnification box.

Figure 7B shows a BDA injection into the left sVc, which extended rostrally to include parts of the lateral reticular formation ventral to sVi. The halo of the injection site included the ventral corner of the caudal sVi. The morphology of several labeled intratrigeminal neurons within the ipsilateral pV/sVo is illustrated in Figure 7A. Retrogradely labeled intratrigeminal neurons were distributed within the ventral portion of pV and extending into the rostral sVo. The labeled neurons ranged from small (Cells 2 and 3) to medium (Cells 1, 4–7), as the somata of these neurons ranged from 19 to 53 μm along their long axis. The labeled neurons were fusiform or multipolar in shape, with 3–5 sparsely branching dendrites. These isodendritic neurons showed no evident pattern in their dendritic orientation within pV. However, their dendrites, to the extent they could be followed, were confined within the nucleus.

Examples of labeled neurons and axon arbors in the contralateral pV/sVo are illustrated in Figure 7C. Only a few retrogradely labeled neurons were observed in this region, and most appeared to be in the border region between the rostral end of sVo and caudoventral pV. These cells varied widely in size. Most of the labeled neurons (Cells 2 and 3) noted in the contralateral pV/sVo, were smaller (long axes = 20 μm) multipolar or fusiform neurons. They displayed sparsely branched dendrites. One, particularly large (long axis = 35 μm), multipolar neuron (Cell 1) was located pV/sVo adjacent to the exiting facial nerve. Several labeled axon terminals were observed in close association with the dendrites of this neuron (arrowheads, Figure 7C, Cell 1).

**Figure 8:**
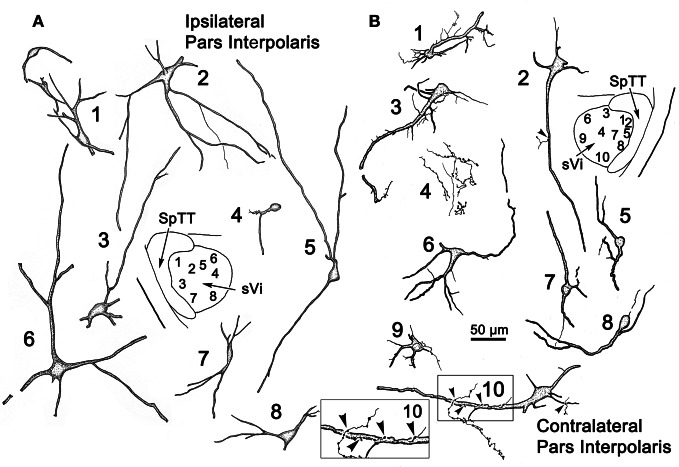
**Retrogradely labeled neurons and anterogradely labeled axons within sVi on the ipsilateral (A) and contralateral (B) side following the injection illustrated in Figure 7B.** The location of the illustrated neurons is shown in the low magnification inserts. An example of the labeled commissural axonal arbors is illustrated in **(B4)**. Close associations (arrowheads) between a labeled cell (Cell 10) and axonal boutons on the contralateral side are shown in the box.

Figure 8 illustrates the morphology of labeled neurons and axon terminal arbors distributed within sVi following the sVc injection (Figure 7B). In the ipsilateral sVi (Figure 8A), retrogradely labeled neurons appeared evenly distributed across this subnucleus. Of particular note were the variations in soma size and dendritic complexity. Both fusiform and multipolar cells were present, with somata ranging in size from 18 to 44 μm in their long axes. Their sparsely branched dendrites were confined within the nucleus. The morphology of several labeled neurons and terminal arbors in the contralateral sVi are catalogued in Figure 8B. Retrogradely labeled neurons tended to be located in the periphery of the contralateral sVi, avoiding the central core of this subnucleus. Like cells in ipsilateral sVi, they displayed considerable variation in soma size and dendritic complexity. Both fusiform and multipolar cells were present, with somata ranging in size from 13 to 31 μm in their long axes. Their sparsely branched dendrites were confined within the nucleus. Cells 1 and 3, located dorsal and adjacent to the SpTT, possessed thick proximal dendrites with unusual fine processes emanating from the parent dendrite. Profile 4 illustrates an anterogradely labeled axonal arbor studded with varicosities, residing in the core of the nucleus. Cell 10, located ventrally, at the periphery of the nucleus, not only projects to the contralateral sVc, it in turn displays a close association (arrowhead) with a labeled axon terminal suggestive of a reciprocal connection between sVc and sVi neurons on either side of the midline.

**Figure 9:**
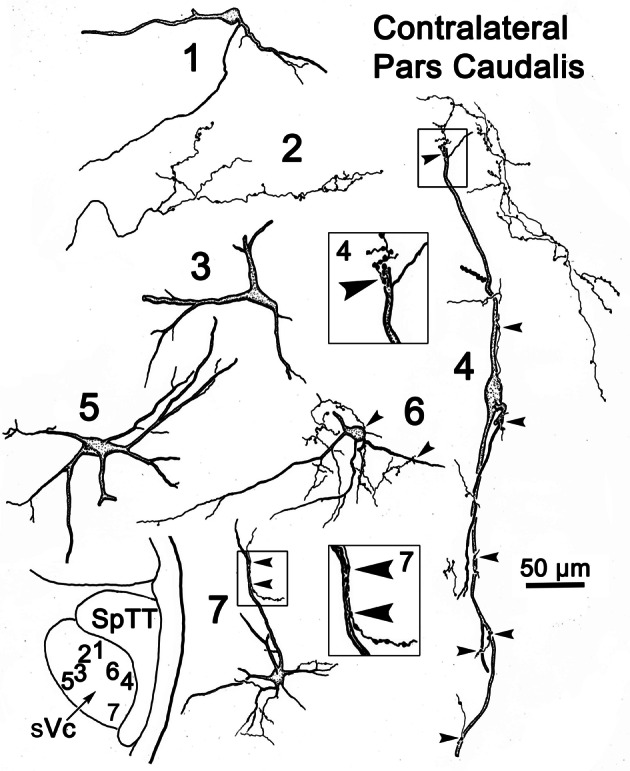
**Retrogradely labeled neurons and anterogradely labeled axons in sVc on the contralateral side following the injection into sVc illustrated in Figure 7B.** Close associations (arrowheads) between labeled cells (Cells 4 and 7) and axonal boutons are shown in the boxes. The location of the illustrated neurons is shown in the low magnification insert.

Figure 9 provides examples of labeled elements in the contralateral sVc following an injection of the opposite nucleus (Figure 7B). The sVc displayed both retrogradely labeled neurons (Cells 1, 3–7), as well as anterogradely labeled axon terminals (Profile 2). Cell 4, lying in lamina I adjacent to SpTT, is oriented parallel to the tract. Anterogradely labeled axons displayed terminal varicosities that were in close association (arrowheads) with cells in layer I (inserts for Cells 4 and 7), suggesting a reciprocal connection between neurons in sVc on either side of the brainstem. Some (Cells 1, 4, and 7), but not all (Cell 6), of the cells with somata in layers I and II confined their dendrites to these laminae. The deeper neurons (Cells 3 and 5) were multipolar, and had no specificity in the direction or orientation of their dendritic branches. Fine, labeled axons in the core of contralateral sVc (Profile 2) had a simple organization and possessed fewer labeled varicosities than those in lamina I (Cell 4).

**Figure 10:**
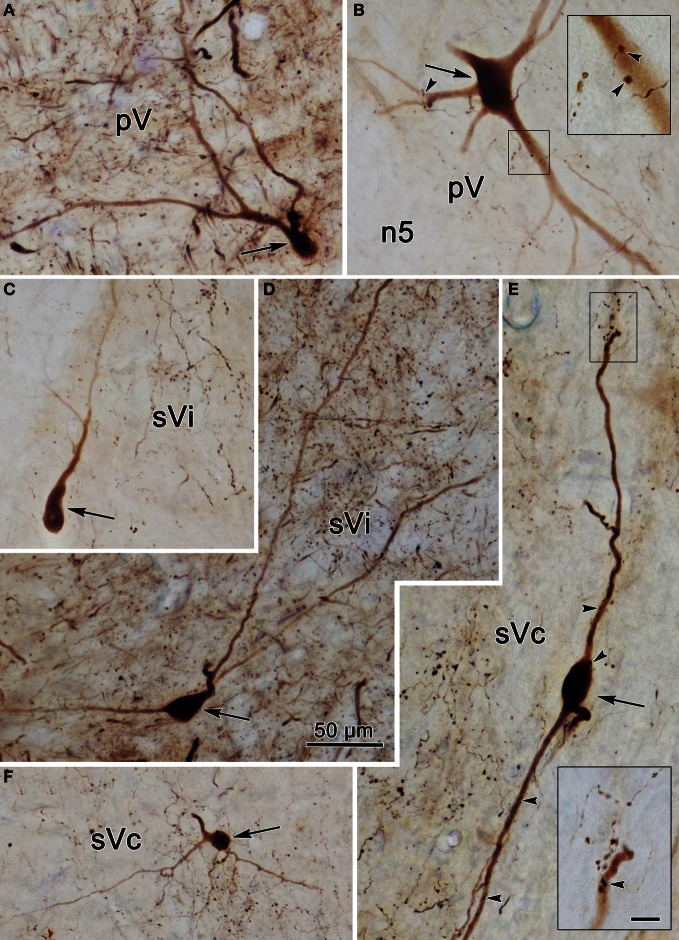
**Photomicrographs of labeled neurons (arrows) and axons within the trigeminal subnuclei following a unilateral injection into sVc (Figure 7B).** Panels **(A,D)** are from the ipsilateral side, while panels **(B,C,E,F)** are from the contralateral side. The box shows the presence of close associations (arrowheads) between labeled axonal boutons and this cell's dendrites. Close associations like those shown in the box were fairly common. Scale in the boxes is 50 μm. Z axis planes used to produce plate: **(A)** = 7, **(B)** = 10 (box = 1), **(C)** = 9, **(D)** = 7, **(E)** = 10 (Box = 2), **(F)** = 8. Scale in **(D)** for **(A–F)**.

Photomicrographs of labeled neurons and axons in the trigeminal nuclei resulting from a unilateral injection into sVc (Figure 7B) are shown in Figure 10. Plate **(A)** shows a labeled multipolar intratrigeminal neuron within the ipsilateral pV/sVo (Figure 7A, Cell 3) with an extensive dendritic field spreading through the nucleus. Plate **(B)** reveals a large, labeled, multipolar neuron in contralateral pV (Figure 7C, Cell 1). Numerous labeled terminals were observed in close association with this cell (arrowheads, insert). Plate **(D)** displays a labeled neuron in ipsilateral sVi whose sparsely branching dendrites extend for long distances within the nucleus. The cells present in contralateral sVi were fewer in number, and more lightly labeled, but labeled terminal arbors were quite common (Figure 10C). Plates (**E,F**) show labeled cells (Figure 8A, Cells 4–5) found within the terminal field made by labeled commissural axons in lamina I and II. Some extended their dendrites parallel to the lamina (E), while other did not (F). Close associations (arrowheads) were present between the labeled boutons (insert) and dendrites.

**Figure 11:**
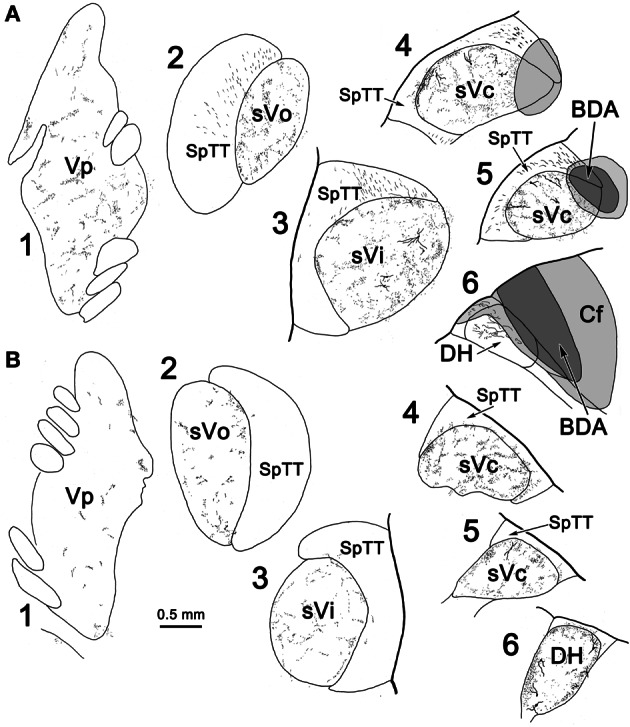
**The pattern of labeling in the ipsilateral (A) and contralateral (B) trigeminal complex following a small injection of sVc.** A BDA injection was placed in sVc at spinal cord level C_2_. It only occupied the dorsal edge of the nucleus, but spread into the cuneate fasciculus (Cf) and the dorsal horn caudal to C_2_(A_4–6_).

Since labeled cells were not observed in contralateral sVc following pV/sVo injections, we initially believed the scattered labeled axons present in this case (Figure 7C) were due to spread of the injection site into sVi. However, analysis of another case (Figure 11) in which a small BDA injection involved the dorsal edge of sVc at C_2_, with spread dorsally into the cuneate fasciculus (Figure 11A_4, 5_) and caudally into the dorsal horn (Figure 11A_6_), showed that terminals were still present in contralateral pV/sVo (Figure 11B_1_). These results suggest that the lack of contralateral retrograde cell label following pV/sVo injections (Figures 1, 2) is more likely due to the relative paucity of terminals present in pV, and that this crossed projection does indeed exist. This small injection was also of interest because while it produced a dense patch of terminals in contralateral sVc in a location mirroring the injection site (Figure 11B_4–6_), a less dense terminal field spread throughout the nucleus. This suggests that both topographic and non-topographic intratrigeminal projections are present. In line with this, retrogradely labeled cells were not confined to the homotopic part of the contralateral sVc, and terminals were seen throughout the contralateral spinal trigeminal nucleus (Figure 11B_2–5_), as well as in the dorsal horn just below C_2_ (Figure 11B_6_). The pattern of ipsilateral label was also of interest. Since the injection site was confined to the dorsal part of the nucleus, and the spinal nucleus is topographically organized with the mandibular branch dorsal, one might have expected that collaterals of the primary afferents would be specifically labeled in isolation within this dorsal mandibular quadrant, throughout the spinal nucleus. In fact, terminal label was seen throughout the spinal trigeminal nucleus (Figure 11A_2–5_) without respect to topography. Widespread terminations were also present in ipsilateral pV (Figure 11A_1_). Presumably, most of these terminals represent the intratrigeminal projections, not primary afferents. Even at the level of the injection, the more diffuse organization of the intratrigeminal connections can be recognized, as labeled terminals and cells were present throughout sVc (Figure 11A_4, 5_).

**Figure 12:**
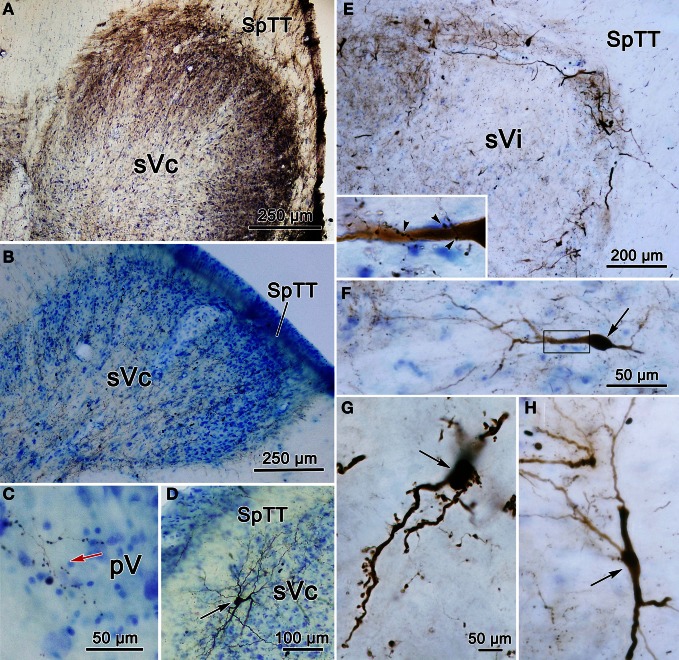
**Photomicrographs of labeled neurons (arrows) and terminals from a BDA injections into sVc are shown (**A–D**) and sVi are shown in (**D,E**). (A)** Is a low magnification of contralateral sVc after a large injection in the spinal portion of sVc. **(B)** Is a low magnification of contralateral sVc after a small injection in the spinal portion of sVc (Figure 11A_4–6_). **(C)** Shows terminals in pV (red arrow) from the same case. **(D)** Shows a retrogradely labeled cell (arrow) in lamina I of ipsilateral sVc ventrolateral to the injection site. **(E)** is a low magnification view that reveals the distribution of labeled cells and axons is much denser in the periphery of contralateral sVi. **(F)** Shows a fusiform cell whose dendrites extend across the nucleus. The boxed area is enlarged in the insert to show labeled axonal boutons making close associations (arrowheads) with this dendrite. **(G)** Shows the dendrites of a particularly well-filled cell that displayed numerous spinous processes. **(H)** Is an example of a cell located near the spinal trigeminal tract, whose dendrites were oriented parallel to the tract. Z axis planes used to produce plate: **(A–D)** = 1, **(E)** = 10, **(F)** = 3, **(G)** = 3, **(H)** = 5. Scale in **(F)** for **(H)**, scale in **(G)** same for insert.

The pattern of label following BDA injections into the spinal portion of sVc is further documented in Figures 12A–D. Figure 12A shows the pattern of label in the contralateral sVc following a large injection that fills sVc. Terminals are present in all laminae, but are densest in lamina I and II. Figure 12B shows the pattern after the small injection illustrated in Figure 11A_4–6_. While far fewer terminals are present, they are still spread through all the laminae. In this case, cells and terminals were also present ipsilaterally in sVc. Figure 12D shows the extensive filling of a lamina I cell located in the maxillary subdivision whose dendrites extended into SpTT. Following this small sVc injection, terminals were present bilaterally at all levels of the spinal nucleus, as well as in pV. A labeled axonal arbor present in contralateral pV is shown in Figure 12C.

**Figure 13:**
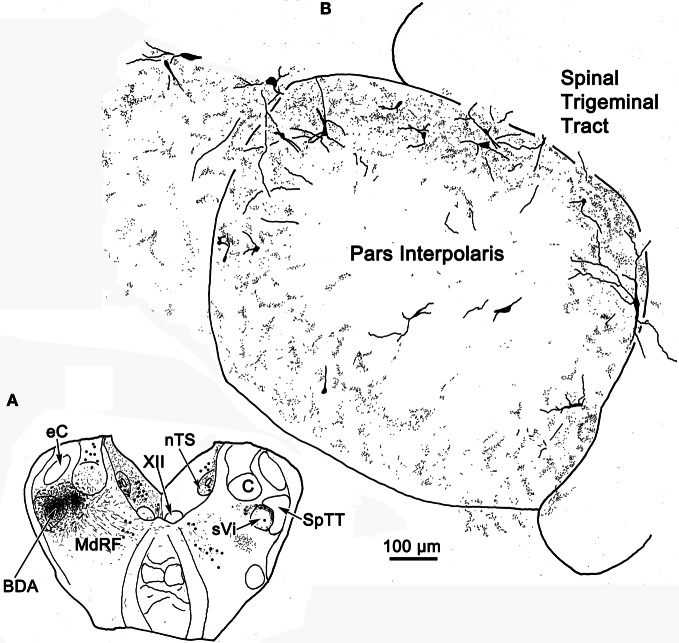
**Robust commissural connections between sVi are shown following a BDA injection (A) at the level of the hypoglossal nucleus (XII).** The injection extended medially into the adjacent medullary reticular formation (MdRF) and resulted in both retrogradely labeled cells (dots) and anterogradely labeled terminals (stipple) in the contralateral MdRF and sVi. **(B)** Anterogradely labeled axon terminals together with retrogradely labeled neurons were most densely distributed around the perimeter of contralateral sVi, avoiding its central region.

**Figure 14:**
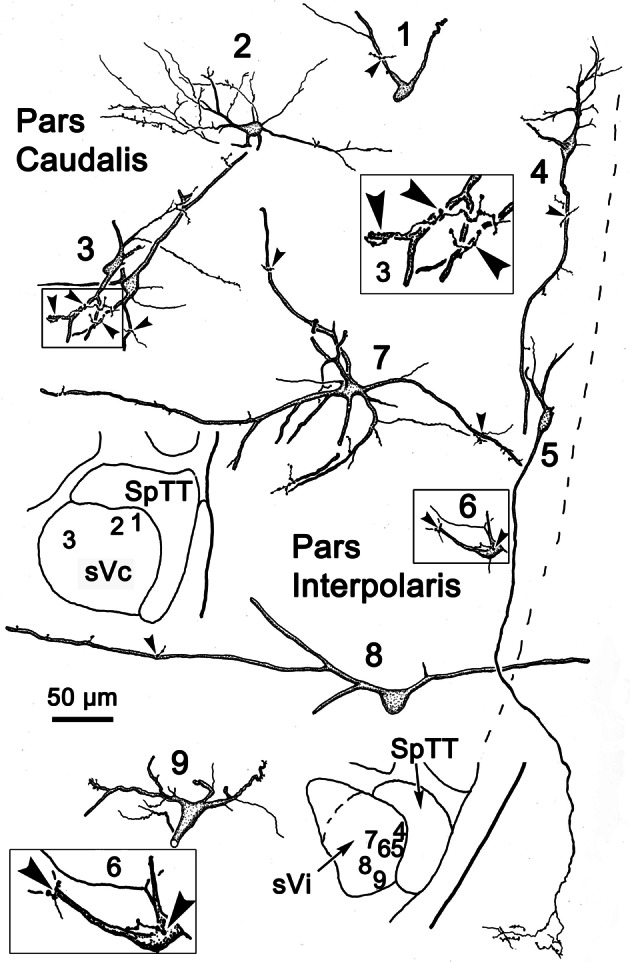
**Following the injection illustrated in Figure 13A, labeled neurons and axon terminals were seen contralaterally in sVc (top) and sVi (bottom).** Cells 1–3 were located in sVc, as shown in the upper low magnification insert. Close associations (arrowheads) with labeled axonal boutons are shown in the higher magnification box for Cell 3. Cells 4–9 were located in sVi, as shown in the lower low magnification insert. Labeled axon terminals formed close associations with some cells, as shown by the high magnification box for Cell 6.

Following an injection of BDA into the left sVi (Figure 13A) that extended medially into the adjacent reticular formation, labeled neurons (dots), and terminals (stipple) were noted within the contralateral sVi. The pattern of labeling in pV/Vo (not illustrated) was similar to that seen after sVc injections. Numerous cells were labeled ipsilaterally in sVc, but only a few were found in contralateral sVc, and these were distributed more rostrally. Sparse terminal label was also present contralaterally in sVc. Examples of the cells located within sVc are shown in Figure 14 (cells 1–3). Their somata were small in size (long axes = 18–20 μm), and they displayed a variety of dendritic field shapes. Cell 1 was found adjacent to the spinal tract (lamina I). It displayed a simple dendritic branching pattern with the labeled branches extending within the lamina toward the tract. Cell 2 (laminae II) is a multipolar neuron with a radiating dendritic field extending across several lamina. The primary dendrites give rise to a number of thin branches. The two deeper cells in laminae IV–V (3) displayed simple dendritic branching patterns that extended superficially.

The pattern of commissural labeling observed after this sVi injection (Figure 13A) is demonstrated in Figure 13B. Many of the intratrigeminal neurons were aligned around the periphery of the nucleus, and were particularly plentiful adjacent to SpTT. These anterogradely labeled terminals overlapped the distribution of labeled neurons in the periphery of sVi. Far fewer labeled elements were present in the central core of sVi. The morphology of the labeled elements in the contralateral spinal trigeminal nucleus following this injection is further illustrated in Figure 14. Cells 4–9 were located within sVi, and showed a wider variety of somatic sizes (long axes = 14–29 μm) and shapes. Cells 4 and 5 were located adjacent to and had dendrites orientated parallel with SpTT. Both had small somata and possessed dendrites with few branches. Cell 4 had curious fine branches emanating from the primary dendrites and soma. Cell 5 extended a single, long, unbranched process into the SpTT. The cells located deeper within the nucleus (Cells 6–9) were highly varied. Cells with both smaller (Cell 6) and larger (Cells 7–9) somata were present. Some displayed very simple dendritic fields (Cell 8), and some had quite complex dendritic fields (Cells 7 and 9). Close associations (arrowheads) between labeled commissural terminals and cells were present in both sVc (inset Cell 3) and sVi (inset Cell 6).

The low magnification photomicrograph in Figure 12E reveals the distribution and overlap of commissural axons terminals and neurons around the periphery of contralateral sVi following the BDA injection illustrated in Figure 13A. In Figure 12, plates (**F–H)** display labeled intratrigeminal neurons from this same case. Some cells (Figure 12H) oriented their dendrites parallel to SpTT, while others (Figure 12F) extended their dendrites perpendicular to it. The insert reveals the existence of close associations between labeled commissural axon terminals (arrowheads) and a labeled cell (Figure 12F). Plate **(G)** shows a portion of a labeled neuron with extensive dendritic filling that revealed the presence of ornate dendritic processes. These were rarely seen, perhaps because they require greater amounts of label in the cytoplasm for observation.

## Discussion

This study provides the first detailed morphological characterization of primate intratrigeminal circuits. It clearly demonstrates the presence of neuronal populations that interconnect the subdivisions of the trigeminal sensory complex both ipsilaterally and, to a lesser degree, contralaterally. Crossed projections yoking the same level of the complex are the most prevalent of the contralateral connections. The intratrigeminal projections originate from a wide variety of fusiform and multipolar neurons located at different depths from the spinal trigeminal tract suggesting that these cells correspond to a variety of physiological classes. In addition, this study supplies suggestive evidence that intratrigeminal axons may even terminate on intratrigeminal cells. The diversity of reciprocal intratrigeminal connections may provide an anatomical substrate for modulation of both trigeminothalamic projection cells and circuits underlying brainstem reflexes in primates.

**Figure 15:**
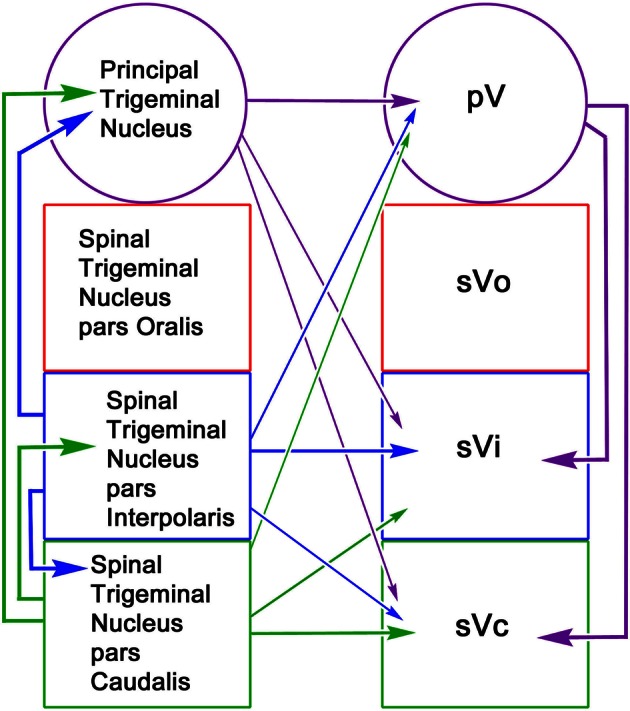
**Schematic of reciprocal intratrigeminal connections demonstrated in this study.** The thickness of the lines indicates the strength of the projection. For simplicity, the connections of the pV/sVo are indicate by arrows to pV, since we did not distinguish between the subnuclei in the experimental material. All possible connections are present. The arrows are color coded based on the location of the cells of origin.

The reciprocal intratrigeminal circuits identified in this study are summarized in Figure 15. Retrograde labeling following pV/sVo injections and anterograde label following spinal trigeminal injections indicates that sVi neurons project both ipsilaterally (thick blue arrow) and contralaterally (thin blue arrow) to pV/sVo, as do sVc neurons (ipsilaterally—thick green arrow) (contralaterally—thin green arrow). This suggests that sVi and sVc neurons have both ipsilateral and contralateral ascending projections that directly influence neuronal activity in pV/sVo. Furthermore, the presence of anterogradely labeled intratrigeminal axon terminals within the contralateral sVc and sVi following pV/sVo injections, and retrogradely labeled cells in pV/sVo following spinal trigeminal injections provides evidence for a descending crossed intratrigeminal pathway originating from pV/sVo neurons (thin purple arrows) that terminates in the spinal trigeminal subdivisions. Both labeled terminals and cells were seen in the contralateral pV/sVo (purple arrow) following unilateral pV/sVo injections, indicating the presence of a considerable commissural connection at the rostral end. Similar homotopic commissural connections were seen with both sVi (blue arrow) and sVc (green arrow) injections. Retrogradely labeled neurons in ipsilateral sVi and pV/sVo following sVc injections provided evidence for both long (thick purple arrow) and short (thick blue arrow) descending ipsilateral pathways to sVc from pV/sVo and sVi, respectively. The anterograde and retrograde label from this same injection indicated that sVc and sVi are interconnected commissurally (thin green and blue arrows). Injections of sVi supported the presence of these projections, and also suggested that pV/sVo provides an ipsilateral descending projection to sVi (thick purple arrow). In general, the strongest projections were ipsilateral, followed by homotopic commissural projections, and heterotopic commissural projections (as indicated by line thickness).

### Technical considerations

The use of BDA in this study confirmed the presence of intratrigeminal connections identified in earlier studies of other species by other methods. This tracer allowed superior morphologic demonstration of the intratrigeminal neurons and axons. It also revealed close associations between BDA labeled axonal boutons and BDA labeled cells that are suggestive of synaptic contacts between intratrigeminal neurons. However, proof of synaptic contact will require ultrastructural examination.

Unfortunately, BDA can also label fibers-of-passage, a problem exaggerated by the structure of the spinal trigeminal nuclear complex, where injections into the subnuclei usually breach a portion of the spinal trigeminal tract. To compensate for this, we also utilized WGA-HRP to demonstrate the projections. The presence of the same pattern of connections visualized with a second tracer characterized by more limited fiber-of-passage uptake strengthens the findings. Nevertheless, many of the BDA labeled axon terminals observed in trigeminal subnuclei ipsilateral to the injection may have resulted from fiber-of-passage labeling of trigeminal primary afferents. Thus, the evidence for ipsilateral trigemino-trigeminal projections rests primarily on the retrograde data, and we did not analyze the ipsilateral anterograde BDA labeling. It should also be noted that injections of sVi may have labeled fibers passing to and from pV and sVi, resulting in spurious anterograde and retrograde labeling. Fiber-of-passage labeling might also be responsible for non-topographic labeling of intratrigeminal cells and axons seen after the small sVc injection (Figures 11, 12).

Spread of tracer outside the injection target is also a confounding factor in interpreting the findings. However, the present findings are reinforced by being demonstrated using both retrograde and anterograde transport. The injections targeting pV/sVo spread to include the middle cerebellar peduncle, the pontine reticular formation, and the parabrachial nuclei. Control injections of the peduncle and of the pontine reticular formation failed to yield a pattern of trigeminal labeling similar to one observed following pV/sVo injections. Thus, spread laterally and medially are unlikely to be a confounding factor in our results. Spread into the parabrachial region is more problematic. Both, sVc (monkey: Wiberg et al., 1987; cat: Ikeda et al., 1984; Panneton and Burton, 1985; Nasution and Shigenaga, 1987; rat: Jacquin et al., 1990b) and sVo neurons (rat: Dallel et al., 2004) are known to target the ipsilateral parabrachial nuclei, including the Kölliker-Fuse nucleus. Although the number and laminar arrangement of cells projecting to the parabrachial region is different than seen with pV injections (cat: Ikeda et al., 1984; Nasution and Shigenaga, 1987; rat: Jacquin et al., 1990b), it is likely that some of the labeled cells observed following pV injections are due to parabrachial spread.

Injections of the spinal trigeminal nuclei often included the reticular formation immediately rostral and/or ventral to the nucleus, and sometimes included the reticular formation just medial to the nucleus. Extension medially caused increased labeling in the contralateral reticular formation, but did change the pattern of trigeminal labeling. Control injections in the medial medullary reticular formation or spinal cord produced little trigeminal labeling. Extension ventrally may have included reticular formation areas related to the trigeminal sensory nuclei. Thus, a portion of the labeled projections may be from this region, but it should be noted that few cells were seen in this region around sVc following pV injections (Figures 1G–I), particularly contralaterally.

Jacquin et al. (1990a) described a large crossed primary afferent projection arising from trigeminal ganglion neurons of the rat that terminated in the contralateral spinal trigeminal nucleus at C_1–2_. While this pattern is superficially similar to the commissural terminal fields noted here in monkey, a crossed primary afferent projection has not been observed in monkeys (Marfurt and Echtenkamp, 1988; May and Porter, 1998) and was not present following control injections of the middle cerebellar peduncle that labeled trigeminal nerve afferents. Finally, the presence of retrograde label in the contralateral sVc is further support for the existence of a trigemino-trigeminal commissural projection.

### The ipsilateral ascending intratrigeminal pathway

Intratrigeminal neurons projecting to ipsilateral pV/sVo were found in sVc, primarily in laminae III and IV, with a few cells in lamina I and II. Only pV/sVo injections labeled numerous pyramidal-shaped neurons whose cell bodies lay in the deeper laminae of sVc. In contrast, the labeled cells located at the same depth in sVi showed a variety of multipolar shapes and sizes. This anatomical difference reinforces the physiological and pharmacological differences observed between sVi and sVc (Bereiter, 1997; Hirata et al., 2000, 2004). In general, the distribution of labeled neurons demonstrated in this study is reminiscent of a distribution pattern shared by other mammalian species, including rats (Falls, 1984a; Jacquin et al., 1990b; Voisin et al., 2002), cats (Carpenter and Hanna, 1961; Stewart and King, 1963; Hockfield and Gobel, 1982; Ikeda et al., 1982, 1984; Panneton and Burton, 1982; Lovick and Wolstencroft, 1983; Somers and Panneton, 1985; Nasution and Shigenaga, 1987), hedgehogs (Ring and Ganchrow, 1983), sheep (Roberts and Matzke, 1971), and other non-human primates (Dunn and Matzke, 1968; Kruger, 1971; Tiwari and King, 1974; Smith, 1975; Kruger et al., 1977; Ganchrow, 1978) suggesting a common mammalian pattern.

Gobel and Purvis (1972) were the first to describe deep fiber bundles traversing the spinal trigeminal nucleus and tract. These intratrigeminal fibers may provide an anatomical substrate for influencing access, processing, and transmission of facial and oral sensations within and between various regions of the trigeminal sensory nucleus in both an excitatory and inhibitory fashion (Furuta et al., 2008; Han et al., 2008). If pV is primarily non-nociceptive in nature, an explanation of a projection from sVc to pV is in order. In fact, physiological studies of spinal trigeminal neurons have identified a variety of response types throughout all subnuclei of the spinal trigeminal complex. For example, low threshold mechanoreceptive responses are common to all the subnuclei (Kruger and Michel, 1962a,b; Kerr et al., 1968; Mosso and Kruger, 1973; Azerad et al., 1982). Furthermore, there is a laminar specific segregation of sensory inputs within sVc (monkey: Price et al., 1976; cat: Hu et al., 1981). Low threshold primary afferent fibers project to laminae III/IV neurons. Nociceptive, thermal and high threshold mechanoreceptive primary afferent fibers project to lamina I, II, and V (Biedenbach, 1973; Mosso and Kruger, 1973; Nord and Ross, 1973; Price et al., 1976; Hu et al., 1981; Azerad et al., 1982). In addition, nociceptive specific, thermal and wide dynamic range neurons have been reported in pV (Eisenman et al., 1963; Yu and King, 1974; Khayyat et al., 1975), and sVo (Eisenman et al., 1963; Davies et al., 1971; Tamarova et al., 1973). Thus, the ascending intratrigeminal pathway, arising from sVc neurons may provide a circuit by which nociceptive signals in lamina I/II provide the nociceptive fields observed in pV/sVo cells, and low threshold mechanical signals in layers III/IV can modify activity of similar neurons in pV/sVo (Denney-Brown and Yanagisawa, 1973; Sessle and Greenwood, 1974; Yu and King, 1974; Khayyat et al., 1975; Greenwood and Sessle, 1976; Shigenaga et al., 1976). Alternatively, Scibetta and King (1969) have suggested that sVc maintains a hyperpolarizing influence on pV neurons, and that the interplay of cross-modal activity between nuclei may be an important feature of the neural mechanisms subserving discrimination and perception of facial stimuli. For example, ascending GABAergic projections from sVi and glutaminergic projections from sVc can modulate the activity of vibrissal mechanoreceptor in rat pV (Furuta et al., 2008). In this fashion, intratrigeminal terminals may modulate transmission of discriminative information about nociceptive and thermal sensation to the sensory thalamus, allowing a more global characterization of stimuli with multimodal characteristics.

### The ipsilateral descending intratrigeminal pathway

The descending intratrigeminal projection has received less attention. Hockfield and Gobel (1982) provided the first anatomical demonstration of a descending ipsilateral intratrigeminal pathway originating from neurons in the rostral spinal trigeminal nuclei. Ikeda et al. (1984) and Nasution and Shigenaga (1987) identified neurons in cat sVo and sVi as the primary source of descending intratrigeminal pathway to sVc. Other studies of the cat descending intratrigeminal pathways (Lovick and Wolstencroft, 1983; Ikeda et al., 1984; Nasution and Shigenaga, 1987) also failed to identify descending intratrigeminal neurons within pV. Similarly, Falls (1984b) identified cells within sVo as the source of the descending intratrigeminal pathway in the rat. In the current study, we demonstrated the presence of a limited numbers of cells in the border region of pV/sVo that are the source of this descending projection to sVc of the monkey. This represents the first demonstration of a descending intratrigeminal pathway in non-human primates. These cells are probably homologous to the neurons observed in the cat and rat sVo. Neurons located at the border between ventral pV and sVo are known to receive convergent polysensory inputs (Dubner, 1967), to have large receptive fields, and to lack somatotopic organization (Eisenman et al., 1963). Thus, these intratrigeminal cells may have properties similar to their downstream targets and they may be conferred by ascending intratrigeminal projections. It is clear that descending projections can both excite and inhibit targeted trigeminal cells (Han et al., 2008), which may be reflected in the variety of intratrigeminal soma sizes observed here.

At present, neither the functional role nor the targets of the descending intratrigeminal pathway are known. The presence of a circuit linking pV/sVo and sVi/sVc suggests that non-noxious stimuli can modify properties of sVc nociceptive cells. Alternatively, it could be argued that the pV/sVo border region may contain multimodal neurons, and the intratrigeminal connections simply link higher order trigeminal neurons with similar characteristics. However, while many multimodal and wide dynamic range neurons are present in sVc, there is little evidence that these cells also have discriminative touch inputs like those seen in pV. One possibility is suggested by the anti-nociceptive connections present in the spinal cord. There, collaterals of dorsal column afferent fibers enter the spinal gray and access circuits that inhibit pain transmission (Wall and Dubner, 1972). Descending intratrigeminal projections include both GABAergic and glycinergic components (Han et al., 2008), so intratrigeminal descending projections may also function as a gate, restricting the access of nociceptive trigeminal signals to higher order tract cells.

### Commissural intratrigeminal connections

A striking finding of the present study was the level of contralateral projections. There is anatomical evidence of reciprocal commissural connections linking the sVc nuclei in cat (Stewart and King, 1963; Hockfield and Gobel, 1982), and in squirrel monkey (Tiwari and King, 1974; Ganchrow, 1978). Commissural neurons in the cat and rat are present in all laminae except II and VI (Hockfield and Gobel, 1982; Jacquin et al., 1990a). In the present study, we saw cells in all layers. In particular, lamina I commissural cells in sVc displayed dendrites extending within lamina I parallel to the spinal trigeminal tract, similar to lamina I neurons with distinctive firing patterns described in the rat (Sedlacek et al., 2007; see also Hockfield and Gobel, 1982). The commissural terminal field was denser in the peripheral lamina as well (Figure 12). This same type of reciprocal organization was seen in the non-laminated caudal sVi following sVi injections (Figure 13). The pattern of connections above and below the interpolaris/caudalis border suggests that the nociceptive cells located here modulate each other across the midline. The present study provides strong retrograde and anterograde evidence for a previously unreported, commissural projection linking the rostral spinal trigeminal nuclei pV/sVo (Figure 15, purple arrow). A portion of the retrograde label could be due to projections to the facial nucleus, but we saw little difference in the pattern of label when injections including or excluding the facial nucleus were compared. This commissural projection suggests that neurons with low threshold mechanoreceptive fields also can be modulated across the midline.

In addition to homotopic commissural projections, we have also observed sparse crossed ascending and descending projections between the nuclei of the trigeminal complex (Figure 15, thin arrows). Lovick and Wolstencroft (1983) demonstrated crossed connections between sVc and sVi of the cat like those observed here. We also observed a descending projection from pV/sVo to the monkey contralateral spinal trigeminal nuclei (sVi and sVc). This contralateral descending projection has not been reported in any species to date. The retrograde data suggest that the source of this projection is a small number of cells located in the ventral pV/sVo border region. The presence of both retrograde and anterograde label argues that this more limited projection is not artifactual, and may simply have been overlooked, previously. The fact that the same region of the pV/sVo appears to be the source of the ipsilateral projection suggests that these two pathways may share the same modulatory function and perhaps underlie conjugate oro-facial reflexes. It is noteworthy that this descending projection is paralleled by an ascending crossed projection to pV/sVo (Figure 15, thin arrows).

A role for the commissural connections observed in this study remains to be determined. A few cells with receptive fields near the midline have been reported to have fields that slightly extend onto the contralateral body surface, but it seems unlikely that the widespread connections we and others have observed serve this purpose. Since this connection does not contribute to the observed receptive fields, it presumably must play a modulatory role. Subthreshold inputs have been shown to play a role in the physiology of somatosensory cortical neurons (Kwegyir-Afful and Simons, 2009) and it is possible that these commissural projections supply such a subthreshold input. Such modulation may be important for perceiving when stimuli move from one side of the face to the other. It is noteworthy that unilateral sensory stimulation can produce bilateral up-regulation of c-fos in the spinal trigeminal nucleus, particularly in lamina I (Nomura et al., 2002). Alternatively, these projections may be inhibitory, and supply the equivalent of an inhibitory surround from the contralateral body surface. Certainly, the wide distribution of these neurons and complex pattern of interconnections argues that they are not confined to a single submodality. In view of these results, it would be of interest to test the receptive fields of trigeminal neurons, while simultaneously probing the other side of the face.

### Functional role in trigeminally evoked conjugate reflexes

Reciprocal intratrigeminal connections may provide a morphological substrate to modulate trigeminal neuronal activity throughout the trigeminal sensory complex. The importance of these circuits in developing an integrated trigeminal signal at the level of the brainstem is emphasized by the fact that proprioceptive signals in the trigeminal mesencephalic nucleus are also relayed by axon collaterals terminating in the principal and spinal trigeminal nuclei (Shigenaga et al., 1990; Luo et al., 1995; Wang and May, 2008). As a group, intratrigeminal connections may allow spatial and/or temporal convergence of peripheral signals within the trigeminal nuclei. Such combined signals may be particularly useful for brainstem reflexes, in contradistinction to the more specific signals relayed to cortex as part of the process of conscious sensory analysis. A single unit electrophysiological study in the cat (Hu et al., 1981) demonstrated that sVc intratrigeminal neurons include both nociceptive and low threshold mechanoreceptive units. The conduction velocity of these cells was slower than for sVc neurons projecting to the thalamus, suggesting they are a separate population. On the other hand, there is evidence in rats that collaterals of sVi trigeminothalamic axons also contribute to the ascending intratrigeminal projection (Jacquin et al., 1989; Jacquin and Rhoades, 1990). As Figure 15 demonstrates, the trigeminal sensory nuclei are extensively interconnected ipsilaterally. Thus, these pathways are poised to coordinate oro-facial reflexes like chewing that require a variety of information from wide regions of the face. In addition, the rich pattern of the commissural connections suggests a route to yoke both sides together; a connection that may be necessary for trigeminally evoked conjugate reflexes like blinking.

### Conflict of interest statement

The authors declare that the research was conducted in the absence of any commercial or financial relationships that could be construed as a potential conflict of interest.
